# Characterization of Novel Aptamers Specifically Directed to Red-Spotted Grouper Nervous Necrosis Virus (RGNNV)-Infected Cells for Mediating Targeted siRNA Delivery

**DOI:** 10.3389/fmicb.2020.00660

**Published:** 2020-04-30

**Authors:** Lingli Zhou, Shaowen Wang, Qing Yu, Shina Wei, Mingzhu Liu, Jingguang Wei, Youhua Huang, Xiaohong Huang, Pengfei Li, Qiwei Qin

**Affiliations:** ^1^School of Life Sciences, Sun Yat-sen University, Guangzhou, China; ^2^Guangdong Laboratory for Lingnan Modern Agriculture, College of Marine Sciences, South China Agricultural University, Guangzhou, China; ^3^Guangxi Key Laboratory of Marine Natural Products and Combinatorial Biosynthesis Chemistry, Guangxi Beibu Gulf Marine Research Center, Guangxi Academy of Sciences, Nanning, China; ^4^Guangxi Key Laboratory of Marine Environmental Science, Guangxi Academy of Sciences, Nanning, China

**Keywords:** aptamer, Cell-SELEX, red-spotted grouper nervous necrosis virus, antiviral activity, targeted delivery, siRNA

## Abstract

Nervous necrosis virus (NNV) causes viral nervous necrosis, the most devastating disease in more than 50 fish species worldwide, with massive mortality rates up to 100%, resulting in great economic losses to mariculture. However, few methods are available for the efficient diagnosis and treatment of viral nervous necrosis. Aptamers are molecular recognition ligands characterized by their remarkably high specificity and affinity, great stability, and ease of synthesis, and have been widely studied in application of disease diagnosis and therapies. In this study, we generated three aptamers against red-spotted grouper nervous necrosis virus (RGNNV)-infected grouper brain (GB) cells using the Cell-SELEX (cell based-systematic evolution of ligands by exponential enrichment) technology. The selected aptamers formed stable stem-loop structures, and could specifically recognize RGNNV-infected GB cells, with calculated dissociation constants (*K*_*d*_) of 27.96, 29.3, and 59.5 nM for aptamers GBN2, GBN10, and GBN34, respectively. They also recognized RGNNV-infected brain tissues. The three aptamers were non-toxic and showed antiviral activities both *in vitro* and *in vivo*. Fluorescence microscopy and flow cytometry also demonstrated that aptamer GBN34 could be efficiently and specifically internalized into RGNNV-infected GB cells. The targeted cellular delivery of aptamer-small interfering RNA (siRNA) conjugates remarkably inhibited RGNNV infection in GB cells. The efficiency of the aptamer-based targeted delivery system was about 75% reduction in infection after 48 h, which was similar to that of transfection. These aptamers have great potential utility in the rapid diagnosis and inhibition of RGNNV infection in mariculture.

## Introduction

Nervous necrosis virus (NNV), one of the most devastating marine viruses, typically causes viral nervous necrosis (VNN) or viral encephalopathy and retinopathy (VER) in the larvae and juveniles of marine and freshwater fish (Souto et al., 2019). In recent years, NNV has spread to more than 50 fish species around Europe, Australia, Martinique, North America, Tahiti, and Asia (Liu et al., 2012; Doan et al., 2017). In severe outbreaks, NNV resulted in high mortality up to 100% within a week (Munday et al., 2002). NNV could be divided into four clade: barfin flounder nervous necrosis virus (BFNNV), striped jack nervous necrosis virus (SJNNV), tiger puffer nervous necrosis virus (TPNNV), and red-spotted grouper nervous necrosis virus (RGNNV) (Nishizawa et al., 1997). Among these, RGNNV is the commonest pathogen associated with fish VNN diseases in Southeast Asian countries and China (Xu et al., 2010). Vacuolization of the brain and retinal tissues can be seen in infected fish, and common symptoms of VNN include abnormal whirling and spiraling, dark body color, and loss of appetite. However, our knowledge of NNV infection and treatment is still limited, and there is no efficient commercial therapeutic regimen for VNN.

Aptamers are functional single-stranded DNA (ssDNA) or ssRNA molecules isolated from random oligonucleotides pools using the selective evolution of ligands by exponential enrichment (SELEX) (Stoltenburg et al., 2007). They could bind to a variety of targets with high specificity and affinity, similar to antibodies (Li et al., 2015a; Fattal et al., 2018; Yu et al., 2019a). However, aptamers have many advantages over antibodies, including their selection *in vitro*, easy synthesis and modification, high reproducibility, long-term stability, small size, and low immunogenicity (Kovacevic et al., 2018). In recent decades, aptamers have begun to compete with antibodies in the rapid detection of specific molecules and therapeutic applications (Shangguan et al., 2006; Tan et al., 2013). For instance, the product Macugen, based on the PEGylated form of the anti-human vascular endothelial growth factor (VEGF) aptamer, was approved in 2004 (United States) and 2006 (Europe) (Chapman and Beckey, 2006). Phase 1 studies for the product development of an RNA aptamer targeting factor IXa were completed in 2006 (Dyke et al., 2006). Many aptamers directed against purified virions, viral proteins, and viral infected cells show great potential utility in viral research, detection, and inhibition (Li et al., 2014, 2015b). For example, a chip-based detection method for hepatitis C virus (HCV) was developed with aptamers directed against the HCV core antigen (Lee et al., 2007). In a previous study, we characterized three aptamers with antiviral activities directed against the RGNNV coat protein (CP) protein (Zhou et al., 2016).

When cells are infected by a virus, their surfaces are modified by the insertion of viral proteins. These modifications and alterations could be important biomarkers of virus-infected cells, and served as therapeutic targets against viral infection (Kim et al., 2018; Zhu and Chen, 2018). However, known biomarkers of specific viral infections are still limited. Nucleic acid probes (aptamers) generated by Cell-SELEX could specifically bind to modified proteins or biomarkers that occurred during viral infection (Zhu and Chen, 2018). Furthermore, the identified aptamers’ targets serve as potential biomarkers of viral infection (Fang and Tan, 2010; Ye et al., 2012). For example, Parekh et al. (2010) selected aptamers for vaccinia virus (VV)-infected cells using Cell-SELEX, and identified A56R, the target of the aptamer, as a potential biomarker of VV infection. Aptamers could also be used in drug delivery. Zhou and Rossi (2012) reported the use of aptamer–siRNA conjugates as a cell-type-specific delivery system for the inhibition of human immunodeficiency virus 1 (HIV-1).

In this study, we applied Cell-SELEX to generate three ssDNA aptamers (GBN2, GBN10, and GBN34) specifically directed against RGNNV-infected grouper brain (GB) cells (RGNNV-GB cells). The binding specificity and affinity of the aptamers for RGNNV-GB cells were determined, and we evaluated the cytotoxicity, histological toxicity, and antiviral activities of the three aptamers, both *in vitro* and *in vivo*. The cell-specific internalization of aptamers was demonstrated, and an aptamer–siRNA delivery system was established to evaluate the antiviral ability against RGNNV infection. This is the first time that aptamers directed against RGNNV-infected GB cells have been generated and applied to siRNA delivery for inhibition of RGNNV infection.

## Materials and Methods

### Ethics Statement

All experimental procedures on animals were approved by the Ethical Committee of College of Marine Sciences, South China Agricultural University. Our animal experiments were conducted in accordance with the guidelines issued by the Ethics Committee of South China Agricultural University. All sections of this report adhere to the ARRIVE (Animal Research: Reporting *in vivo* Experiments guidelines for reporting animal research).

### Fish, Cells, Virus, and Reagents

Healthy fry (3–4 cm) and juveniles (8–10 cm) of the grouper *Epinephelus coioides* were purchased from a fish farm in Hainan Province, China. Before the experiment, the fish were temporarily cultured in an air-pumped laboratory recirculating seawater system (2.5% salinity) for 2 weeks.

Grouper brain cells, which are permissive to RGNNV, were propagated in Leibovitz’s L15 medium (Gibco, Grand Island, NY, United States) supplemented with 10% fetal bovine serum (FBS; Life Technologies, Carlsbad, CA, United States) at 25°C, as described previously (Huang et al., 2011). RGNNV is maintained in our laboratory. The 50% tissue culture infectious dose (TCID_50_) of the RGNNV stock in the GB cells was determined as described previously (Reed and Muench, 1938).

### Initial Random ssDNA Library and Primers for Cell-SELEX

The synthetic initial ssDNA library (Sigma-Aldrich, St. Louis, MO, United States) consisted of a central randomized sequence of 50 nucleotides (nt) flanked by two primer hybridization sites (5′-GACGCTTACTCAGGTGTGACTCG-N_50_-CGAAGGACGCAGATGAAGTCTC-3′). A fluorescein isothiocyanate (FITC)-labeled forward primer (5′-FITC-GACGCTTACTCAGGTGTGACTCG-3′) and a biotinylated reverse primer (5′-biotin-GAGACTTCATCTGCGTCCTTCG-3′) were used for the PCRs.

### Cell-SELEX

The SELEX procedure was performed essentially as described previously, with modifications (Li et al., 2015b). GB cells were grown to 100% confluence in 60 mm cell culture dishes (Corning Inc., Corning, NY, United States), infected with RGNNV at a multiplicity of infection (MOI) of 1, and incubated at 25°C for 24 h. The initial ssDNA library (10 nmol) was denatured by heating at 95°C for 5 min, cooled on ice for 10 min, and then dissolved in 1000 μl of binding buffer (5 g/L glucose, 10% FBS; Life Technologies) containing 1.0 g/L bovine serum albumin (Solarbio, Beijing, China), 0.1 mg/ml yeast tRNA (Invitrogen, Carlsbad, CA, United States), and 5 mM MgCl_2_. The ssDNA mixture was then incubated with RGNNV-infected cells for 60 min at 4°C. After being washed with washing buffer (10 mM Tris–HCl, 5 g/L glucose, 9 g/L NaCl, and 5 mM MgCl_2_), the bound ssDNAs were eluted from the collected cells by incubation at 95°C for 5 min. After centrifugation, the supernatant containing the ssDNAs was collected for PCR. The amplified products were denatured by heating at 95°C for 5 min and then renatured by cooling immediately on ice for 5 min. The sense ssDNAs were separated from the biotin-conjugated antisense strands using streptavidin-coated Sepharose beads (Promega, United States) as previously described (Paul et al., 2009). The collected sense ssDNAs were used in the next round of selection. To evolve aptamer candidates with high affinity and specificity, the incubation time was reduced, the washing strength was increased, the number of RGNNV-GB cells was gradually reduced, and counter selection was incorporated into the third and subsequent selection cycles. For counter selection, we incubated normal GB cells with the sense ssDNAs and collected the supernatant for the next round of selection. The 10th enriched ssDNA library was amplified, cloned, and sequenced. The candidate aptamer sequences were aligned and clustered with ClustalW2 (Chenna et al., 2003). The final aptamers were predicted with the MFold program^1^ (Yang et al., 2013).

### Specificity Analysis of Aptamer Candidates Recognizing RGNNV Infected GB Cells by Flow Cytometry

Flow cytometry was used to monitor the enrichment of the selection library and to measure the specific binding of each candidate aptamer to RGNNV-GB cells. Predenatured FITC-labeled aptamer candidates (200 nM) were cooled on ice for 5 min and then incubated with 5 × 10^5^ RGNNV-GB cells in binding buffer for 1 h. After incubation, the cells were washed three times with phosphate-buffered saline (PBS) and suspended in 400 μl of PBS. Fluorescence was measured with a FACS Calibur flow cytometer (BD Biosciences, United States) by counting 20,000 events. FITC-labeled aptamer candidates incubated with normal GB cells were used as the negative controls.

### Specific Binding of Aptamers to RGNNV-GB Cells Detected With Fluorescence Microscopy

For fluorescent imaging, the carboxytetramethylrhodamine (TAMARA)-labeled aptamers (200 nM) were denatured at 95°C for 5 min, and cooled on ice for 5 min. They were then added to RGNNV-GB cells in 35 mm glass bottom dishes (Cellvis, catalog number D35-14-1-N). After incubation at 4°C for 1 h in a darkroom, unbound aptamers were washed off, and 4% paraformaldehyde was added to the cells to fix them. The aptamers incubated with uninfected GB cells and SGIV-infected GB cells were used as controls. The cells were imaged on a Leica DMRXA fluorescence microscope (Leica, Wetzlar, Germany).

### Specific Binding of Aptamers to RGNNV-Infected Brain Tissue Detected With Fluorescence Microscopy

Each individual grouper, *E. coioides*, was starved for 24 h and then intraperitoneally injected with 40 μl of 10^7^ TCID_50_/ml RGNNV. Grouper fry injected with PBS served as the controls. Each group contained 30 grouper fry. After 1 week, the brain tissues of each group were collected and fixed in 10% neutral-buffered formalin. The fixed tissue sections were stored before the subsequent experiment. The frozen tissue sections were incubated with TAMARA labeled aptamers (200 nM) at 4°C for 1 h in a darkroom. After the samples were washed with PBS, the tissues were imaged with a Leica DMRXA fluorescence microscope. Tissue sections incubated with the TAMARA-labeled initial library were used as the controls.

### Measurement of Aptamer Affinity

The procedure used to measure the binding affinity of the aptamers has been reported previously by Li et al. (2015a). The mean fluorescence intensity of the FITC-labeled aptamers bound to RGNNV-GB cells was used to calculate their equilibrium dissociation constants (*K*_*d*_) according to the equation *Y* = *B*_*max*_*X*/(*K*_*d*_ + *X*) with SigmaPlot. *B*_*max*_ indicated the maximum mean fluorescence intensities of target cells bound with aptamers. *Y* indicated the mean fluorescence intensities of target cells bound with aptamers at corresponding concentrations (*X*). Results from three independent experiments are presented as the mean ± SD.

### Trypsin Treatment of RGNNV-Infected GB Cells

Red-spotted grouper nervous necrosis virus-infected grouper brain cells (5 × 10^5^) were digested with 1 ml of 0.25?% trypsin (Thermo Scientific HyClone) at 28°C for 10 min. Then, medium supplemented with 10% FBS was added to inhibit the activity of trypsin. After washing with PBS three times, trypsin-treated cells were incubated with FITC-labeled aptamers. Fluorescence was assessed with a FACScan cytometer by counting 20,000 events as described previously (Li et al., 2015a).

### Cytotoxicity and Histological Toxicity Analyses

For the cytotoxicity analysis, GB cells were seeded in 96-well plates for 18 h. Each aptamer was serially diluted to produce a concentration gradient (0, 1, 10, 100, and 1000 nM), and added into GB cells in 96-well plates for 48 h. The cytotoxicity of the aptamers was measured by cell proliferation reagent WST-1 (Roche, Mannheim, Germany)-based colorimetric assay, according to the manufacturer’s instructions. Three independent experiments were performed for each set of experimental conditions.

For the histological toxicity analysis, healthy grouper (*E. coioides*) were starved for 24 h before the experiment. Each grouper was then injected intraperitoneally with each aptamer (GBN2, GBN10, and GBN34; 1000 nM). There were two control groups: control 1, normal grouper received no injection; control 2, grouper injected with PBS. Each treatment group contained 10 fry. Mortality was recorded daily until no further mortality occurred. The histological toxicity of aptamer GBN34 using hematoxylin–eosin (H & E) staining was analyzed as described previously (Zhou et al., 2016).

The toxicity of aptamers was also assessed by biochemical parameters, including alanine aminotransferase (ALT), aspartate aminotransferase (AST), and creatinine (Cr). The liver of the experimental fish was weighed maximum up to 0.1 g and homogenized in 2 ml of 0.5 M (pH 7.4) Tris–HCl buffer. The mixture was then centrifuged at 8000 rpm for 15 min at 4°C and the supernatant was collected for the analysis of ALT and AST. The activities of AST and ALT were measured using a transaminase assay kit (Transaminase C II-test, Wako, Osaka, Japan), according to the manufacturer’s instructions. Blood was gathered from the caudal veins and kept in clean tubes, left for 2 h, and then centrifuged at 3000 rpm, 4°C, for 15 min, followed by serum separation. The serum Cr was measured using Creatinine Assay Kit (Abcam, Cambridge, United Kingdom), according to the manufacturer’s instructions.

### Inhibition of RGNNV Infection *in vitro*

The inhibition of RGNNV infection by selected aptamers *in vitro* was analyzed as previously described (Liang et al., 2013), with a few modifications. GB cells were seeded in 24-well plates for 18 h. Each aptamer (200 nM) and RGNNV (MOI = 0.5) were then added to the GB cells at 28°C for 24 h. To assess the dose effects of the aptamers on RGNNV infection, different concentrations (100, 250, 500, and 1000 nM) of aptamer (GBN2) were added to the GB cells. There were two control groups: 1: GB cells incubated with RGNNV (MOI = 0.5) alone, 2: GB cells incubated with the mixtures of initial random library (1000 nM) and RGNNV (MOI = 0.5). After infection with RGNNV at 28°C for 12 and 24 h, respectively, we imaged the cell morphology and cytopathic effects (CPEs; vacuolation) with light microscopy. We also collected cells from each well to measure the relative expression of CP mRNA with quantitative real-time reverse transcription (RT)-PCR (qRT-PCR), using previously described methods (Huang et al., 2015). The results of three independent experiments were averaged. Another batch of cells were collected and lysed with low-salt lysis buffer for Western blot (WB). Proteins were transferred to polyvinylidene fluoride (PVDF) membranes (Bio-Rad, CA, United States) and further incubated with the anti-CP antibodies. The LumiGlo Chemiluminescent Substrate System (KPL, Guildford, United Kingdom) was used for protein detection.

To determine the antiviral activities of aptamers to RGNNV-infected cells, each aptamer was added to the infected cells (12 hp.i.). After aptamer treatment for 12 and 24 h, respectively, we collected cells to measure the relative expression of CP mRNA with qRT-PCR and CP protein level with WB, as described above.

### Inhibition of RGNNV Infection *in vivo*

Healthy *E. coioides* fry (3–4 cm) were starved for 24 h before the experiment. Each grouper was then injected intraperitoneally with a mixture of 40 μl of 10^7^ TCID_50_/ml RGNNV and each aptamer (GBN2 and GBN34; 1000 nM). There were five control groups: control 1, grouper fry injected with a mixture of the same amounts of RGNNV and the initial random library; control 2, grouper fry injected with RGNNV alone; control 3, grouper fry injected with aptamer GBN2 alone; control 4, grouper fry injected with aptamer GBN34 alone; control 5, grouper fry injected with PBS. Each treatment group contained 30 fry. Mortality was recorded daily until no further mortality occurred.

### Cell-Specific Internalization Study of GBN34

A fluorescence-microscopy-based analysis of cell-specific internalization was performed as previously described, with some modification (Xiao et al., 2008). GB cells were seeded in 35 mm glass bottom dishes (Cellvis) for 18 h before infection. RGNNV (MOI = 1) was then added to the GB cells, which were then cultured at 28°C for 8 h. After being washed twice with PBS, RGNNV-GB cells were incubated with FITC-labeled aptamer GBN34 (200 nM) in L15 medium at 28°C for 2 h. A quick wash with NaOH (200 mM) was used to remove any cell-surface-bound aptamer before the internalization study. The cells were then washed twice with PBS and visualized under a Leica DMRXA fluorescence microscope. RGNNV-GB cells incubated with the FITC-labeled ssDNA library (200 nM) and uninfected GB cells incubated with the FITC-labeled aptamer GBN34 (200 nM) were used as the control.

For the flow-cytometric analysis of cell-specific internalization, GB cells were seeded and incubated in six-well plates for 18 h. RGNNV (MOI = 1) was then added to infect the GB cells, and incubated at 28°C for 8 h. After the RGNNV-GB cells were washed twice with PBS, they were incubated with FITC-labeled aptamer GBN34 (200 nM) in L15 for 2 h at 28°C. FITC-labeled aptamer GBN34 (200 nM) incubated with RGNNV-infected cells at 4°C served as the control group. The cells were then washed twice with PBS. Fluorescence in the target cells was assessed with a FACScan cytometer by counting 20,000 events. The results of three independent experiments were averaged.

### Assembly of Aptamer–siRNA Conjugate

The aptamer–siRNA conjugate was assembled as previously described, with some modification (Chu et al., 2006). The biotin-labeled siRNA (Bio-siRNA) and control Bio-siRNA (Bio-NC) were designed and synthesized by RiboBio (Guangzhou, China). The following RNA oligonucleotides were used in this study:

control siRNA (siRNA-NC) forward: 5′-CAAGUAAA GACUGCCUAAU-3′
Reverse: 5′-AUUAGGCAGUCUUUACUUG-3′
RGNNV CP siRNA #1 forward: 5′-UCGUCGGCGUA GUAAUCGCTT-3′
Reverse: 5′-GCGAUUACUACGCCGACGATT-3′
RGNNV CP siRNA #2 forward: 5′-CCUCGACUGUAA CUGGAUUTT-3′
Reverse: 5′-AAUCCAGUUACAGUCGAGGTT-3′

The biotin-labeled aptamer (GBN34) was denatured at 95°C for 5 min and cooled on ice for 5 min. The aptamer–siRNA conjugates were assembled by mixing the biotin-labeled siRNA and aptamer, and streptavidin at the molar ratio of 2:2:1 for 15 min. The conjugation of the aptamer and siRNA to streptavidin was confirmed with a gel-shift analysis.

We examined the silencing effects of siRNAs by measuring the relative viral CP mRNA expression by qRT-PCR, using previously described methods (Huang et al., 2015). GB cells were seeded in 24-well plates for 18 h. The cells were then transfected with siRNAs or Bio-siRNAs at a concentration of 100 or 200 nM using Lipofectamine 2000, according to the manufacturer’s instructions. After transfection for 24 h, RGNNV (MOI = 0.5) was added to the GB cells for culture. GB cells transfected with Bio-NC (200 nM) were used as the control. We collected the cells from each well to measure the relative CP mRNA expression with qRT-PCR, using previously described methods (Huang et al., 2015). The results of three independent experiments were averaged.

### Cellular Delivery of Aptamer–siRNA Conjugates for Inhibition of RGNNV Infection

The aptamer–siRNA conjugate, at a final concentration of 50 nM (100 nM aptamer; on average, two aptamers/conjugate), and RGNNV (MOI = 0.5) were added to GB cells for culture. GB cells to which aptamer (200 nM) and RGNNV (MOI = 0.5) were added were used as a control. Another control consisted of GB cells to which only RGNNV (MOI = 0.5) was added. To evaluate the efficiency of delivery, we used a matched group in which GB cells were transfected with 50 nM conjugate for 24 h before RGNNV (MOI = 0.5) was added to the cells. GB cells in other wells were transfected with Bio-NC to test the effects of the transfection reagents. We collected the cells from each well to measure the relative CP mRNA expression with qRT-PCR, using previously described methods (Huang et al., 2015). The results of three independent experiments were averaged.

To determine the antiviral activities of aptamer–siRNA conjugates to RGNNV-infected cells, the conjugate was added to the RGNNV-infected cells (12 hp.i.). After aptamer treatment for 24 and 36 h, we collected cells to measure the relative expression of CP mRNA with qRT-PCR.

## Results

### Cell-SELEX Targeting RGNNV-GB Cells and the Enrichment of Aptamer Candidates

**FIGURE 1:**
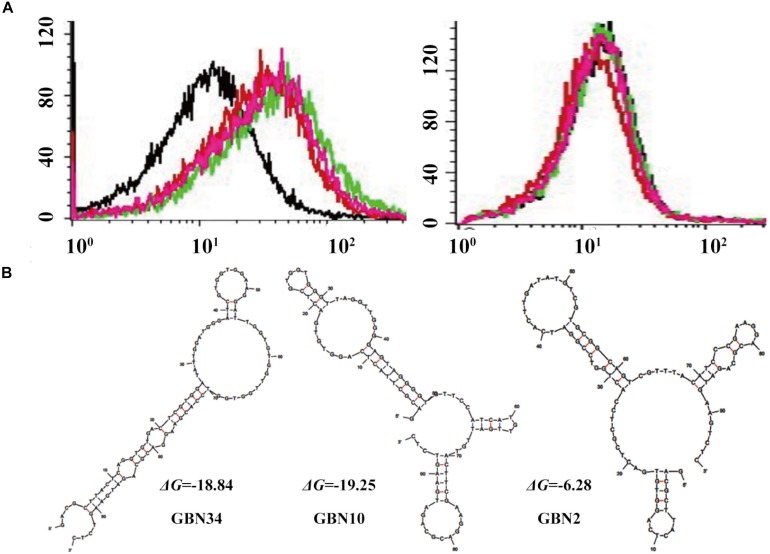
Selection of three aptamers that specifically recognized RGNNV-GB cells by cell-systematic evolution of ligands by exponential enrichment (Cell-SELEX) technology. **(A)** Binding of FITC-labeled DNA pools to RGNNV-infected GB cells was enhanced after more rounds of selection (left). Black, initial library; red, 9th round of selection; green, 10th round of selection; pink, 11th round of selection. FITC-labeled DNA pools bound to uninfected GB cells served as a control (right). **(B)** Predicted secondary structures of aptamers (GBN2, GBN10, and GBN34). The stabilities of the aptamer secondary structures were calculated as the free energy values (Δ*G*).

We used flow cytometry to monitor the enrichment of the ssDNA pools. As shown in Figure 1A, the fluorescence intensity of the ssDNA pools bound to RGNNV-GB cells increased as the SELEX progressed, whereas there was no obvious increase in the fluorescence intensity of the ssDNA pools incubated with uninfected GB cells. The shift in the fluorescent signal showed that the 10th ssDNA pool bound most strongly to the RGNNV-GB cells. Therefore, we cloned and sequenced the highly enriched 10th ssDNA pool.

Three candidate aptamers, GBN2, GBN10, and GBN34 (Table 1), were isolated from the sequenced pools for further analysis, based on the results of a multiple sequence alignment. Prediction of the secondary structures of the selected aptamers revealed that the three aptamers all formed stable stem-loop structures, and GBN2, GBN10, and GBN34 displayed Δ*G* values of −6.28, −19.25, and −18.84 kJ/mol, respectively (Figure 1B).

**TABLE 1:** Identification of ssDNA aptamers.

Aptamer	Central randomized sequences	Frequency
GBN34	TGGATATTGGATGGGATCGTGGTGGAAGGATTGGTGTGGTTGGTGGTCCA	46%
GBN2	CTCCACTGGTCCGGATCACTTGATATGTCGTGCGGCAGTCGTTTACATCC	30%
GBN10	TGGTGGGTTAGGTTGGGGAGTAGGGGTGTTCCATCATGTTGATTGTACTT	24%

*Frequency indicates the percentage of the 11th selected pool comprised by each aptamer.*

### Characterization of Aptamers for RGNNV-GB Cells and RGNNV-Infected Brain Tissues

**FIGURE 2:**
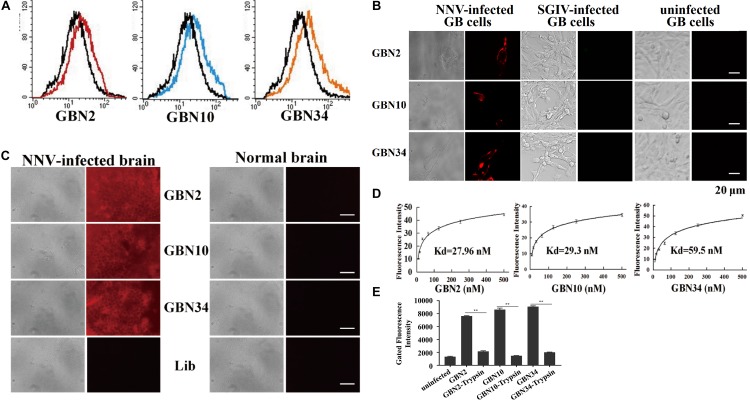
Binding specificity and affinity of three aptamers for RGNNV-infected GB cells. **(A)** Increased fluorescence intensity revealed the binding ability of each FITC-labeled aptamer (red, GBN2; blue, GBN10; orange, GBN34) for RGNNV-infected GB cells. Each FITC-labeled aptamer was tested against uninfected GB cells as controls (black). **(B)** Specific binding of each TAMARA-labeled aptamer to RGNNV-infected GB cells, SGIV-infected GB, or uninfected GB cells. Left, bright-field microscopy; right, fluorescence microscopy. Scale bars indicate 20 μm. **(C)** Fluorescent images of the three TAMRA-labeled aptamers binding to infected brain tissues. Normal brain tissues and the TAMRA-labeled initial library were used as controls. Left, bright-field images; right, fluorescence microscopic images. Scale bars indicate 50 μm. **(D)** Affinity of each aptamer for RGNNV-infected GB cells. Binding affinities of GBN2, GBN10, and GBN34 for RGNNV-infected GB cells were 27.96, 29.3, and 59.5 nM, respectively. **(E)** Changes in fluorescence intensities of aptamers that bound to RGNNV-infected GB cells treated with trypsin.

The binding properties of each aptamer (GBN2, GBN10, or GBN34) to RGNNV-GB cells were analyzed further. In each case, the increased fluorescence intensity detected with flow cytometry indicated that the aptamer bound to RGNNV-GB cells. Fluorescence microscopy also showed that each aptamer bound to RGNNV-infected GB cells and brain tissue, but not to uninfected GB cells, SGIV-infected GB cells, or uninfected grouper brain tissue, which was consistent with the results of flow cytometry (Figures 2A–C). The calculated dissociation constants (*K*_*d*_) for GBN2, GBN10, and GBN34 for RGNNV-GB cells were 27.96, 29.3, and 59.5 nM, respectively (Figure 2D). To determine the target molecules recognized by these aptamers, trypsin treatment was used to remove the membrane protein of cells. As indicated by the fluorescence intensity, the binding of the three aptamers to RGNNV-infected cells was almost completely abolished by trypsin treatment (Figure 2E).

### Cytotoxicity and Histological Toxicity of the Aptamers

**FIGURE 3:**
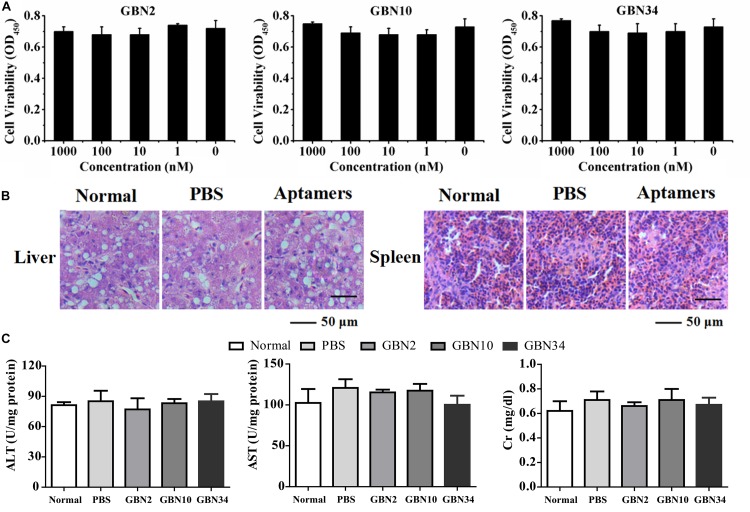
Cytotoxicity and histological toxicity assays. **(A)** Cytotoxicity of each aptamer serially diluted to a concentration gradient (0, 1, 10, 100, or 1000 nM) was measured with a colorimetric assay based on the cell proliferation reagent WST-1. All three aptamers showed no significant cytotoxic effects on GB cells, even at high concentrations of 1000 nM. Results of three independent experiments were averaged. **(B)** Histological toxicity analysis of GBN34 also showed no obvious pathological changes in the liver or spleen tissues of the tested fish. **(C)** Analysis of activity of ALT, AST of liver, and serum Cr. (**P < 0.01; *P < 0.05).

The cell viability analysis with a WST-1-based colorimetric assay showed that the three aptamers had no significant cytotoxic effect on GB cells, even at rather high concentration of 1000 nM (Figure 3A). The toxicity test performed *in vivo* showed that the cumulative mortality rate remained at 0% and the fish were still healthy after 10 days of each aptamer injection. A histological toxicity analysis of GBN34 also proved no obvious pathological changes in the liver or spleen tissues of the fish tested (Figure 3B). Hepatic enzymes (ALP and AST) and serum Cr of aptamers-injected groups displayed no significant difference to that of controls (Figure 3C). Thus, all three aptamers were non-toxic both *in vitro* and *in vivo*.

### Antiviral Effects of Aptamers Against RGNNV Infection *in vitro*

**FIGURE 4:**
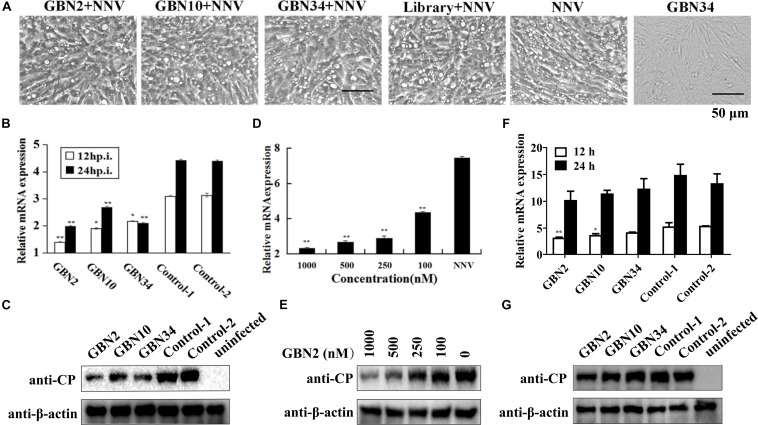
Inhibition of RGNNV infection by selected aptamers *in vitro*. **(A)** Cell morphology and cytopathic effects (vacuolation) after infection with RGNNV, with or without added aptamer. **(B,C)** Relative CP mRNA expression **(B)** and CP level **(C)** were measured to determine the antiviral activity of the aptamer in cultured GB cells. **(D,E)** Aptamers inhibited RGNNV infection in a dose-dependent manner showed by relative CP mRNA expression **(D)** and CP level **(E)**. **(F,G)** Relative CP mRNA expression **(F)** and CP level **(G)** was determined to assess the curative effect of the aptamers in cultured GB cells. (**P < 0.01; *P < 0.05).

The aptamers could inhibit RGNNV infection (Figure 4). As shown in Figure 4A, the cells grew well for 24 h when cultured with aptamer (GBN34) only. When exposed to RGNNV with/without the ssDNA library, extensive CPEs were observed. However, the few CPEs that appeared in the cells incubate with mixtures of aptamer (GBN2, GBN10, or GBN34) and RGNNV. Similarly, compared with the controls, qRT-PCR and WB showed a reduction in the relative expression of viral CP mRNA and protein level in the cells treated with both aptamer and RGNNV. Among the three aptamers, GBN2 showed the strongest capacity to reduce CP mRNA expression, suggesting that this aptamer inhibits RGNNV infection (Figures 4B,C). The inhibition of RGNNV infection by the aptamer was dose-dependent (Figures 4D,E). Aptamers showed little antiviral effects against RGNNV-infected cells at 12 h post supplement of aptamers (24 hp.i.) (Figures 4F,G).

### Antiviral Effects of Aptamers Against RGNNV Infection *in vivo*

**FIGURE 5:**
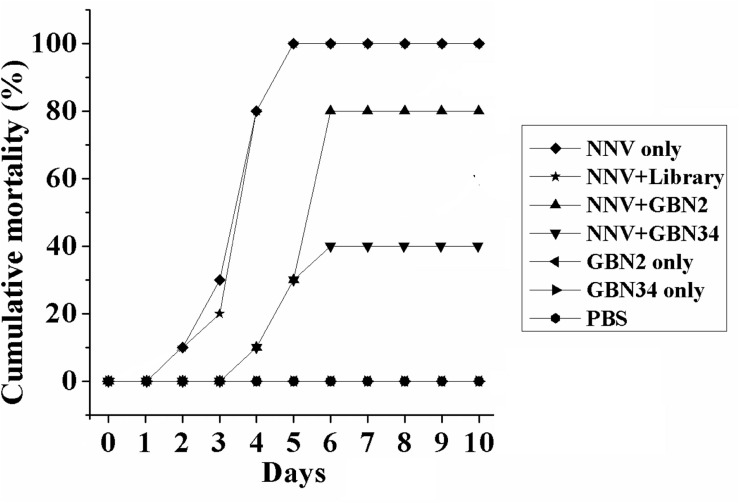
Inhibition of RGNNV infection by aptamers *in vivo*. Values indicate the cumulative mortality in each group of groupers (*E. coioides*) during the 10-day experimental period after each treatment. The selected aptamers inhibited RGNNV infection in groupers.

The aptamers also inhibited RGNNV infection *in vivo*. Ten days after injection, the groupers in the control groups, treated with PBS or aptamer, grew and were healthy, and no fish died. When injected with RGNNV only, 10% of the groupers died on day 2, and all the groupers died after 5 days post infection. The diseased groupers displayed several clinical signs, including whirling and spiraling. This was also observed in the group treated simultaneously with RGNNV and the random ssDNA library. However, when the groupers were simultaneously injected with RGNNV and an aptamer, no fish died within the first 3 days. The cumulative mortality reached 80 or 40% on day 6 in the groups treated with RGNNV premixed with GBN2 or GBN34, respectively. No fish died between days 7 and 10. These results indicated that the generated aptamers could inhibit RGNNV infection in groupers (Figure 5).

### Cell-Specific Internalization of Aptamer

**FIGURE 6:**
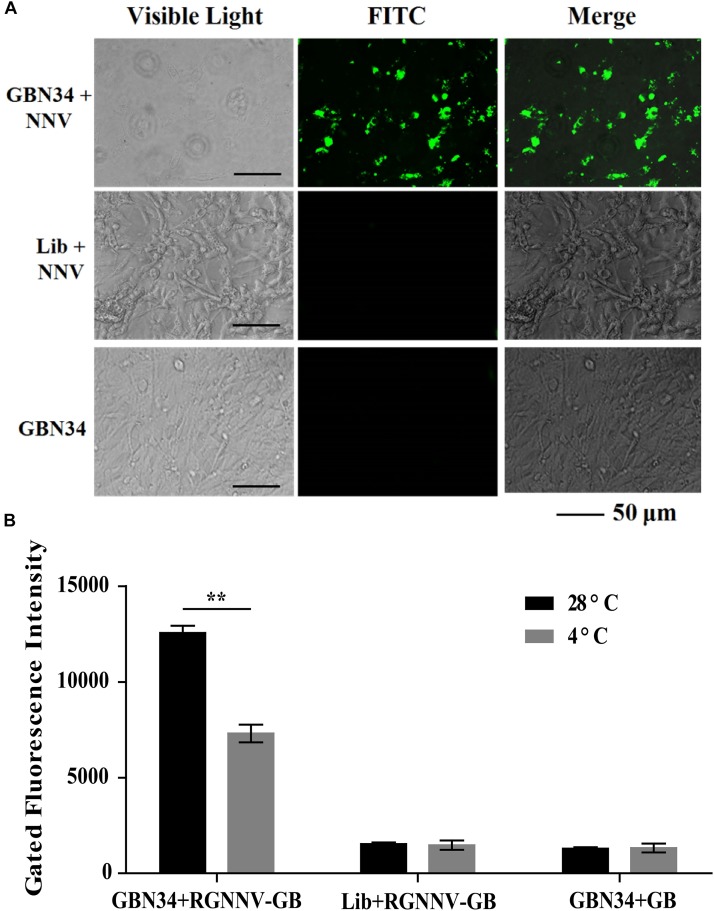
Cell-specific internalization of aptamer GBN34. **(A)** Fluorescent images of FITC-labeled GBN34 after incubation with RGNNV-infected cells for 2 h at 28°C. Scale bars indicate 50 μm. **(B)** Internalization analysis of GBN34. Fluorescence values of FAM-labeled GBN34 incubated with RGNNV-infected cells at 28°C were much more than at 4°C, with the specific active internalization of aptamer GBN34 into RGNNV-infected cells.

The specific internalization of aptamer by RGNNV-infected cells was observed with fluorescence microscopy. After incubation with the FITC-labeled aptamer GBN34 (200 nM) for 2 h, we observed the fluorescent signal of FITC inside the RGNNV-GB cells. In contrast, no obvious fluorescent signal was detected inside the GB cells incubated with the FITC-labeled random ssDNA library or uninfected GB cells (Figure 6A).

As incubation temperature of 4°C could end cell activity and block the possible active endocytosis, it made aptamers only bind to the targets on the cell surface (Figure 2B). Flow cytometry results showed that fluorescence values of FAM-labeled GBN34 incubated with RGNNV-infected cells at 28°C were much more than at 4°C (Figure 6B). The results showed the specific active internalization of aptamer GBN34 into RGNNV-infected cells.

### Cellular Delivery of Aptamer–siRNA Conjugate to Inhibit RGNNV Infection

**FIGURE 7:**
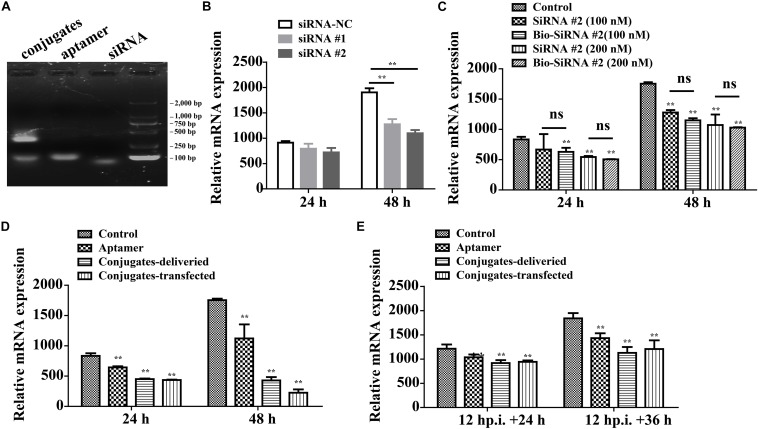
Aptamer conjugate for the delivery of functional siRNA. **(A)** Gel-shift analysis confirmed the conjugation of the Bio-aptamer and Bio-siRNA to streptavidin. **(B)** Gene silencing efficiency of siRNA for CP. **(C)** The biotin label did not significantly affect the silencing efficiency. **(D)** Inhibition of RGNNV infection by aptamer–siRNA conjugate. Compared with the control cells incubated with RGNNV alone, the relative CP mRNA expression decreased by 36.0% at 48 hp.i. in the cells exposed to mixtures of RGNNV and aptamer (100 nM) and by 75% in the cells exposed to mixtures of RGNNV and the aptamer–siRNA conjugates (50 nM). The efficiency of the delivery system (75% reduction in CP mRNA expression at 48 hp.i.) was slightly inferior to that of a transfection system, which reduced CP mRNA expression by 87.2% at 48 hp.i. **(E)** The conjugates showed relatively less antiviral effect against RGNNV-infected cells (24 hp.i.). (**P < 0.01; *P < 0.05).

On average, each aptamer–siRNA conjugate contained a streptavidin molecule, two Bio-siRNA molecules, and two Bio-aptamer molecules. A gel-shift analysis confirmed the conjugation of the aptamer and siRNA to streptavidin (Figure 7A).

The potency of siRNA alone in silencing viral CP expression was determined. siRNA at a final concentration of 100 or 200 nM significantly reduced the relative CP mRNA expression, at 48 hp.i. The biotin label did not significantly affect the silencing efficiency (Figures 7B,C).

Compared with the control cells incubated with RGNNV alone, the relative CP mRNA expression decreased by 36.0% at 48 hp.i. in the cells exposed to mixtures of RGNNV and aptamer (100 nM), and by 75% in the cells exposed to mixtures of RGNNV and the aptamer–siRNA conjugates (50 nM). These results demonstrate that the cellular delivery of the aptamer–siRNA conjugate contributed to the inhibition of RGNNV infection. The efficiency of the delivery system (reducing infection by 75% at 48 hp.i.) was only slightly inferior to that achieved with a transfection system, which reduced CP expression by 87.2% at 48 hp.i., indicating that the aptamer is potentially an excellent targeted carrier (Figure 7D). The conjugates also showed antiviral effects against RGNNV-infected cells (Figure 7E).

## Discussion

Viral nervous necrosis is a serious disease causing mass mortality in larval and juvenile fish worldwide, resulting in great economic impact on the aquaculture of many fish species (Gye and Nishizawa, 2019). However, no biomarkers are available for the development of effective diagnosis or efficient drugs for the treatment against NNV (Liu et al., 2015). Therefore, the development of rapid diagnostic and therapeutic methods for VNN is urgently required. Aptamers are attractive molecular recognition ligands for use in both diagnostics and therapeutics because of their numerous advantages, including high specificity and affinity for targets, strong stability, no toxicity, and ease of synthesis (Syed and Pervaiz, 2010; Alshaer et al., 2018; Bouvier-Müller and Ducongé, 2018). Here, we applied Cell-SELEX to generate three aptamer-targeted RGNNV-infected GB cells. The aptamers displayed anti-RGNNV activities *in vitro* and *in vivo*. Furthermore, as the aptamers could be internalized into RGNNV-GB cells, they had potential utility as delivery agents. To the best of our knowledge, this is the first study to report aptamers directed against RGNNV-infected grouper cells.

Aptamers fold into distinct and stable structures, which underlie their ability to bind to and interrupt the functions of their targets. The diverse secondary and advanced structures of aptamers allow them to bind to a wide variety of targets, including small-molecule compounds, proteins, viruses, and whole cells, by hydrogen bonds, the stacking of aromatic rings, van der Waals forces, or a combination of these (Syed and Pervaiz, 2010). In the present study, all three aptamers were predicted to form stable stem-loop structures, with Δ*G* values of −6.28, −19.25, and −18.84 kJ/mol for GBN2, GBN10, and GBN34, respectively. They also specifically recognized RGNNV-GB cells. The diversity of aptamers’ secondary structures results in complex distinct three-dimensional structures, leading to different affinities of the aptamers for their targets (Stoltenburg et al., 2007). The three aptamers showed high affinities for RGNNV-GB cells, with calculated dissociation constants (*K*_*d*_) of 27.96, 29.3, and 59.5 nM for GBN2, GBN10, and GBN34, respectively. The affinities at the nanomolar level are competitive with those of many antibodies and are consistent with the results of previous studies (Toh et al., 2015).

As a new generation of molecular recognition elements, aptamers have been widely used to replace traditional molecular recognition elements, such as antibodies, in disease diagnostic systems (Wang et al., 2011; Zhou et al., 2014). Because virus-infected cells or cancer cells are often associated with deviations in certain molecular signatures compared with uninfected or normal cells, the detection and measurement of these marker molecules are key steps in developing diagnostic techniques. Li et al. (2016) developed an aptamer-based enzyme-linked aptasorbent assay to detect Singapore grouper iridovirus (SGIV) infection using an aptamer targeting SGIV-infected grouper spleen cells. In the present study, the three aptamers recognized not only RGNNV-GB cells, but also RGNNV-infected grouper brain tissue, whereas they did not bind to uninfected cells or brain tissue, demonstrating their excellent potential utility in RGNNV diagnosis.

The results concluded that the aptamers’ target molecules of GBN2, GBN10, and GBN34 might be directly or indirectly related to surface proteins anchored on the RGNNV-infected GB cell membrane, as they were trypsin-sensitive. Cell membrane proteins have important effects in various physiological functions, for example, the abnormal expression of membrane proteins in cancer cells. After being infected by virus, cell membrane is modified by the insertion of viral proteins or variation of host proteins. These insertion and alterations could be important biomarkers in viral pathogenesis studies (Kim et al., 2018; Zhu and Chen, 2018; Yu et al., 2019b). However, it was always difficult to isolate such membrane proteins because of the low solubility and low abundance. The greatest advantage of aptamers in disease diagnosis is that the complex and time-consuming process of marker identification can be circumvented. Many recent studies have demonstrated that aptamers selected with Cell-SELEX are useful tools for biomarker discovery and identification (Fang and Tan, 2010). For example, Yu et al. (2019c) identified major capsid protein (MCP) as a potential biomarker of grouper iridovirus-infected cells using aptamers. Liu et al. (2009) successfully used aptamers against liver cancer cells to develop sensitive aptamer-nanoparticle strip biosensors for cancer cells, in the absence of any explicit biomarker. Simaeys et al. (2014) identified membrane protein stress-induced phosphoprotein 1 (STIP1) as the target of applied aptamer TOV6, which was generated against ovarian clear cell adenocarcinoma TOV-21G. Aptamer S3 was selected to target NPC 5-8F cells. Using mass spectrometry, membrane protein CD109 was identified as the target of aptamer S3, and a further study identified CD109 as a biomarker of nasopharyngeal carcinoma (Jia et al., 2016). These results suggested that aptamers had a great potential in cell biomarker identification. Although a large number of studies have concentrated on the isolation and identification of RGNNV, its clinical symptoms, and epidemiology, there have been few studies of the biomarkers of RGNNV infection. To address these limits, we would develop the novel molecular probes aptamers, in the combination of two-dimensional gel electrophoresis (2D-GE) and mass spectrometry (MS), to isolate and identify the cell-specific membrane biomarkers for the specific recognition and identification of biomarkers in RGNNV-infected cell membrane in the future study. Based on their ability to distinguish RGNNV-infected and -uninfected cells, the three aptamers developed here could be used to identify the signature molecules of RGNNV infection.

Most antivirus medicines display antiviral effects by disturbing the process of virus infection (Liu et al., 2019). Wang et al. (2016) identified (-)-epigallocatechin-3-gallate as a potential agent for blocking infection of grass carp reovirus. Liang et al. (2013) reported aptamers that targeted rabies virus (RABV)-infected cells, displaying anti-RABV activity. In our study, the three aptamers also inhibited RGNNV infection. An *in vitro* experiment indicated that the inhibition of RGNNV infection by the aptamers was dose-dependent. Among the three aptamers, GBN2 showed the greatest ability to reduce the expression of CP mRNA in GB cells. An *in vivo* experiment showed that GBN2 and GBN34 reduced the cumulative mortality of infected fish by 20 and 60%, respectively. The little antiviral effects of aptamers against RGNNV-infected cells at 24 hp.i. proved that aptamers could display the antiviral effects against RGNNV during the viral infection stage of binding and entering to host cells, but not virus replication in host cells. The antiviral mechanism of aptamers could be that aptamers specifically bind to virus-infected cells with high affinity and interact with their targets, usually biomarkers of viral infection. Interaction of aptamer and its target results in the conformation change of their targets and blocking their functions (Fang and Tan, 2010; Yu et al., 2019c). Therefore, aptamers act as potential antiviral agents. Cytotoxicity and histological toxicity analyses also showed that our aptamers are non-toxic, consistent with previous reports (Syed and Pervaiz, 2010; Ye et al., 2012). These results suggest that GBN2 and GBN34 can be used in RGNNV-targeting therapies. More systemic studies should be done to explore the possible antiviral mechanisms of aptamers against RGNNV in the future.

For clinical applications, drugs should only enter diseased cells, thereby limiting the adverse effects of their non-specific activities in normal cells (Sawyers, 2004). Aptamers that bind cells and are subsequently internalized are highly desirable tools that specifically deliver molecules into cells (Chu et al., 2006), and aptamers have been successfully used to deliver siRNAs, toxins, anticancer drugs, and so on (Zhou et al., 2017). Farokhzad et al. (2004) demonstrated that aptamers directed against prostate-specific membrane antigen (PSMA) were internalized. Based on this result, Bagalkot et al. (2007) established a quantum dot–aptamer–doxorubicin conjugate that could be used as a targeted cancer therapy. Chu et al. (2006) used streptavidin to conjugate the biotin-labeled anti-PSMA aptamer to siRNA, and used this system to inhibit the target gene in cultured cells. Fluorescence microscopy and flow cytometry demonstrated that the aptamer GBN34 was efficiently and specifically internalized by RGNNV-GB cells at a constant rate for 4 h. Considering its non-toxicity, GBN34 has great potential utility in targeted intracellular delivery. Therefore, we assembled an aptamer–siRNA conjugate by mixing biotin-labeled siRNA and the aptamer, conjugated with streptavidin, in a molar ratio of 2:2:1 for 15 min. The conjugation to streptavidin was confirmed with a gel-shift analysis. An antiviral study in GB cells showed that the cellular delivery of the aptamer–siRNA conjugate contributed to the inhibition of RGNNV infection. The efficiency of the delivery system was similar to that of a transfection system (75% reduction in infection at 48 hp.i.). However, with the delivery system, no additional treatment was required, such as transfection with Lipofectamine 2000. These results suggest that GBN34 is an efficient delivery agent. The use of the aptamer–siRNA conjugate may provide a convenient and efficient way to inhibit RGNNV.

## Conclusion

In this study, we generated three aptamers targeting RGNNV-infected GB cells by Cell-SELEX method. These aptamers specifically recognized RGNNV infection *in vitro* and *in vivo* with high affinity in the nanomolar range. Antiviral analysis showed that the aptamers could inhibit RGNNV infection both *in vitro* and *in vivo*. The selected aptamers were also specifically internalized by RGNNV-GB cells, showing their potential utility as delivery agents. Our findings provide new insight into the applicability of aptamers developed with Cell-SELEX to RGNNV diagnosis and inhibition.

## Data Availability Statement

The raw data supporting the conclusion of this article will be made available by the authors, without undue reservation, to any qualified researcher.

## Ethics Statement

The animal study was reviewed and approved by the Ethical Committee of College of Marine Sciences, South China Agricultural University.

## Author Contributions

QQ and PL conceived and directed this study, and revised the manuscript. LZ and PL performed the experiments, analyzed the data, and wrote the manuscript. SWW, QY, SW, and ML performed the experiments and analyzed the data. JW, YH, and XH analyzed the data and revised the manuscript. All authors approved the manuscript to be published.

## Conflict of Interest

The authors declare that the research was conducted in the absence of any commercial or financial relationships that could be construed as a potential conflict of interest.

## Footnotes

1 http://mfold.rna.albany.edu/?q=mfold/DNA-Folding-Form
